# Phenolic Profiles of Ten Australian Faba Bean Varieties

**DOI:** 10.3390/molecules26154642

**Published:** 2021-07-30

**Authors:** Joel B. Johnson, Daniel J. Skylas, Janice S. Mani, Jinle Xiang, Kerry B. Walsh, Mani Naiker

**Affiliations:** 1 School of Health, Medical & Applied Sciences, CQUniversity Australia, Bruce Hwy, North Rockhampton, QLD 4701, Australia; janice.mani@gmail.com (J.S.M.); k.walsh@cqu.edu.au (K.B.W.); m.naiker@cqu.edu.au (M.N.); 2 Australian Export Grains Innovation Centre, North Ryde, NSW 2113, Australia; daniel.skylas@aegic.org.au; 3 Faculty of Food & Bioengineering, Henan University of Science & Technology, Luoyang 471023, China; xjl5013@haust.edu.cn

**Keywords:** phenolic acids, flavonoids, *Vicia faba*, functional food

## Abstract

Although Australia is the largest exporter of faba bean globally, there is limited information available on the levels of bioactive compounds found in current commercial faba bean varieties grown in this country. This study profiled the phenolic acid and flavonoid composition of 10 Australian faba bean varieties, grown at two different locations. Phenolic profiling by HPLC-DAD revealed the most abundant flavonoid to be catechin, followed by rutin. For the phenolic acids, syringic acid was found in high concentrations (72.4–122.5 mg/kg), while protocatechuic, vanillic, *p*-hydroxybenzoic, chlorogenic, *p*-coumaric, and trans-ferulic acid were all found in low concentrations. The content of most individual phenolics varied significantly with the variety, while some effect of the growing location was also observed. This information could be used by food processors and plant breeders to maximise the potential health benefits of Australian-grown faba bean.

## 1. Introduction

Faba bean (*Vicia faba* L.) is reported to be the third most important legume crop [1], with over 5.4 million tonnes being harvested globally in 2019 [1]. After China and Ethiopia, Australia is the third-largest faba bean producer in the world and the largest exporter, providing at least one-third of the internationally traded crop volume [2]. The crop is primarily consumed in China, Southeast Asian countries, and countries in the Middle East.

In recent years, there has been an increasing interest in faba bean due to its nutritional content [1,3] and health-benefitting properties [4]. It is a valuable source of protein, containing twice the protein content of cereal grains in addition to a number of essential amino acids [1]. Furthermore, reported health benefits include improving cardiovascular health [5], providing anti-obesity effects [5,6], anti-cancer activity [5], anti-inflammatory activity [7] and inhibiting xanthine oxidase [8]. This has led to an interest in using faba bean or its isolates in functional food applications [3]. One of the major groups of health-benefiting compounds found in faba bean are phenolics [7].

Due to genotypic variations, which can influence the phenolic and flavonoid biosynthetic pathways [9], the phenolic content may vary significantly between different varieties of grains and pulses [10]. Hence, there has been recent interest in identifying faba bean varieties with high levels of phenolic content. For example, Valente et al. [11] and Valente et al. [12] profiled the phenolic content and antioxidant of seven European faba bean varieties, finding that the levels of total and individual phenolic acids and flavonoids differed significantly between varieties. Similarly, Baginsky et al. [13] found clear differences in the phenolic composition of 10 faba bean varieties grown in Chile, although it should be noted that this study was performed on immature seed material. Another earlier study highlighted the range in total phenolic contents and antioxidant activity among 13 Tunisian faba bean cultivars [14]. However, despite Australia’s international importance as a faba bean producer, there are few comparative studies reporting the phenolic contents of faba bean varieties commonly grown in this country.

Nasar-Abbas et al. [15] reported on the phenolics found in one Australian faba bean variety, while Siah et al. [16] and Siah et al. [5] investigated two and three varieties, respectively. Siah et al. [17] studied the phenolic content and antioxidant activity in five Australian faba bean varieties. Recently, Johnson et al. [18] reported on the antioxidant activity, total anthocyanin content, and total phenolic content of 10 Australian faba bean varieties, although individual phenolic acids or flavonoids were not investigated. This work aims to aid in filling this knowledge gap, presenting phenolic profiles on the 10 faba bean varieties studied by Johnson et al. [18]. It is hoped that this will provide further insight into the nutritional and health-benefitting properties of common Australian faba bean cultivars, as well as providing valuable information on the extent of their genotypic variation present in terms of phenolic acid and flavonoid biosynthesis pathways.

## 2. Results and Discussion

### 2.1. Total Phenolic Contents

**Figure 1:**
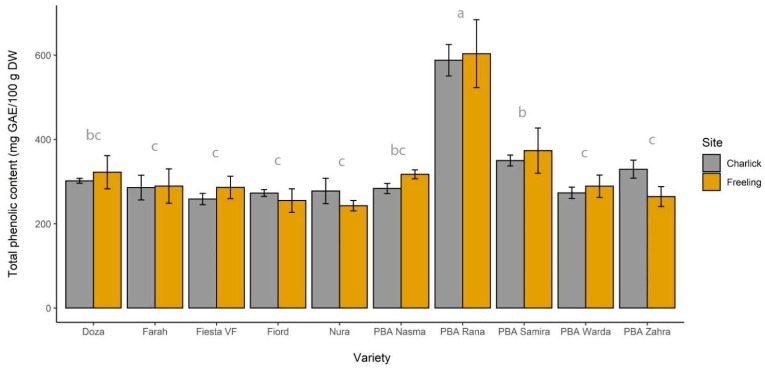
Total phenolic content of the 10 faba bean varieties at each of the growing sites (*n* = 3 field replicates for each bar). The letters (a–c) above each variety show the statistical significance of an ANOVA by variety averaged across both growing locations. Varieties with the same letter were not statistically different from one another at α = 0.05.

The total phenolic content (TPC), as measured by the Folin–Ciocalteu assay, ranged from 258 to 570 mg GAE/100 g (DW) in the different faba bean varieties (Figure 1). The PBA Rana variety contained a significantly higher total phenolic content compared to all remaining varieties. A two-way ANOVA revealed that the site had no significant impact on the total phenolic content (*p* < 0.05), with samples grown at Charlick having a mean TPC of 322 ± 96 mg GAE/100 g (*n* = 30), compared to 324 ± 107 mg GAE/100 g for samples grown at Freeling (*n* = 30). In addition, there was no significant interaction between growing site × variety (*p* > 0.05).

### 2.2. Phenolic acid Profiling by HPLC

**Figure 2:**
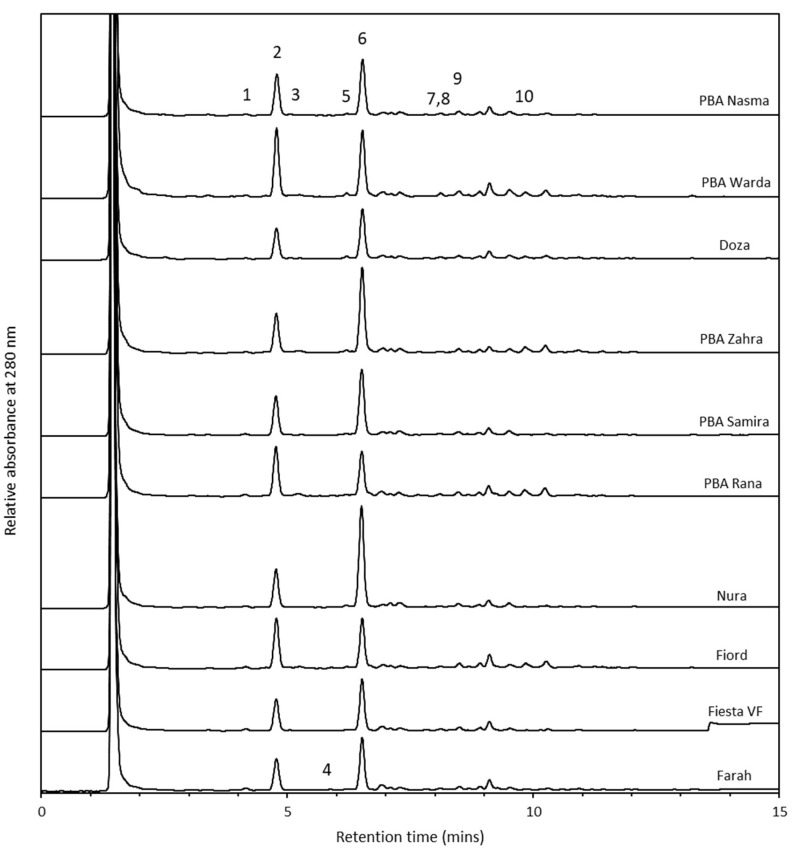
HPLC chromatograms of the phenolic compounds in the 10 faba bean varieties. The compounds indicated are (1) protocatechuic acid, (2) catechin, (3) chlorogenic acid, (4) *p*-hydroxybenzoic acid, (5) vanillic acid, (6) syringic acid, (7) *p*-coumaric acid, (8) vitexin, (9), *trans*-ferulic acid, (10) rutin.

A total of 10 phenolic compounds were identified in the faba bean extracts (Figure 2), comprising of four hydroxybenzoic acids, three hydroxycinnamic acids and three flavonoid-related compounds (Table 1). The following compounds were all determined as being either absent or below the limit of detection: gallic acid, gentisic acid, isovanillic acid, caffeic acid, sinapic acid, cinnamic acid and quercetin-3-glucoside.

The hydroxybenzoic acids found here (protocatechuic acid, *p*-hydroxybenzoic acid, vanillic acid and syringic acid) have all been previously reported from faba bean, as have two of the hydroxycinnamic acids (*p*-coumaric acid and *trans*-ferulic acid) [19,20,21]. In addition, several of these phenolic acids have been found in faba bean pods [11]. The concentrations of free *p*-coumaric and ferulic acids found here were similar to that reported by Liu et al. [21] in Canadian faba bean, although only one variety was included in that study. However, the concentration of syringic acid was much higher compared to previous studies [21,22]. Although the reason for this difference is unclear, it is worth noting that levels of this compound varied significantly between different genotypes (Table 1) and that the soil microbiota composition can also have a significant impact [23]. Similarly, although chlorogenic acid does not appear to have been previously found in faba bean seed, it is produced in the roots of this plant [24]. The low concentrations and high levels of environmental variability may account for its absence in previous work.

The levels of catechin reported here (ranging from 191–297 mg/kg for different varieties) were at the lower range of concentrations reported by Baginsky et al. [13] in the immature seed material of 10 Chilean faba bean varieties, with catechin contents varying between 85–978 mg/kg.

Although vitexin is more commonly known to occur in mungbean [25], it has been previously reported from faba bean using UHPLC-ESI-QTOF-MS-based metabolic profiling [26,27], although it was not quantified. Similarly, although rutin (quercetin-3-rutinoside) does not appear to have been previously reported from faba bean seed, numerous other types of quercetin glycosides have been found in this matrix [8,11]. In addition, rutin has been reported from the flower tissue of several faba bean genotypes, indicating that the synthetic pathways for the production of this compound do occur in faba bean [9].

A two-way ANOVA revealed that the content of all constituents, apart from chlorogenic acid and rutin, varied significantly with variety (Table 1). For most of these compounds, the highest concentrations were found in PBA Rana, which also contained the highest total phenolic content (Figure 1). However, Nura showed the highest levels of syringic acid and total hydroxybenzoic acids.

Similarly, the two-way ANOVA demonstrated that in the case of the 2017 growing season, the site had a significant impact on the content of protocatechuic acid, vanillic acid, chlorogenic acid, vitexin and rutin, as well as on the total amounts of hydroxybenzoic acids and hydroxycinnamic acids (Table 2). For both hydroxybenzoic acids (protocatechuic acid and vanillic acid), samples grown at the Freeling site showed higher contents; while for chlorogenic acid and the flavonoids catechin and rutin, the Charlick samples showed higher concentrations.

For the 2017 growing season, no significant effects of growing location were found for *p*-hydroxybenzoic acid, syringic acid, *p*-coumaric acid, *trans*-ferulic acid, catechin or the total amount of flavonoids. Significant interactions were found between the variety and growing site for several parameters, namely vanillic acid, syringic acid, *trans*-ferulic acid and the sum of hydroxybenzoic acids. It should be noted that the present study investigated only one growing season; hence the results found here may not be generalizable across a wider range of seasons and locations.

There appears to be limited previous literature investigating the impact of growing site and variety × growing site interaction on phenolic acid content in faba bean; however, Mpofu et al. [28] found a significant impact of growing location on six phenolic acids in wheat. In contrast, Oomah et al. [29] found very little impact of growing location on the total phenolic content of 13 faba bean genotypes grown at two locations in Canada.

### 2.3. Principal Component Analysis and Correlation Analysis

**Figure 3:**
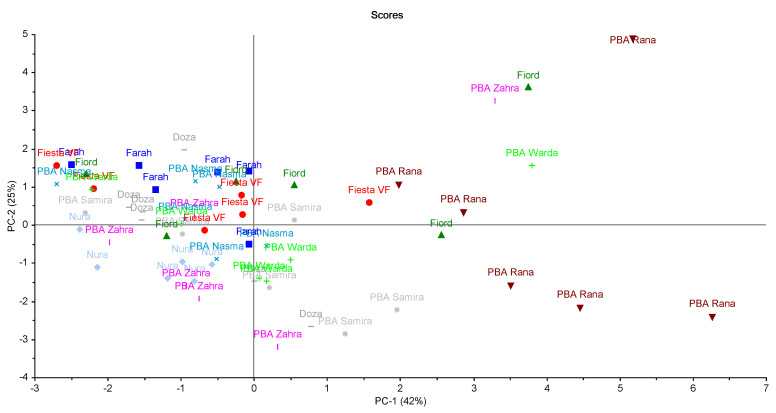
Scores plot showing the results of the principal component analysis performed on the normalised phenolic data. Each faba bean variety is indicated by a different symbol color and shape.

Overall, PBA Rana appeared to have the highest levels of most phenolic acids and flavonoids, possessing a distinct chemical profile compared to the other varieties. This observation was supported by the results of the principal component analysis performed on the normalised phenolic data, which revealed that most samples of PBA Rana were clustered toward the lower right of the scores plot, separated from the majority of other genotypes (Figure 3). Examination of the loadings plot revealed that this corresponded with higher concentrations of catechin and protocatechuic acid, and lower concentrations of syringic acid. In addition, this concurred with previous work highlighting the unique bioactive profile of this genotype [18].

In contrast, the variety Nura was clustered toward the lower left of the scores plot, with syringic acid also weighted on this region of the two PCS. The remaining varieties were more or less clustered around the centre of the scores plot, indicating a relatively similar phenolic composition between them.

**Figure 4:**
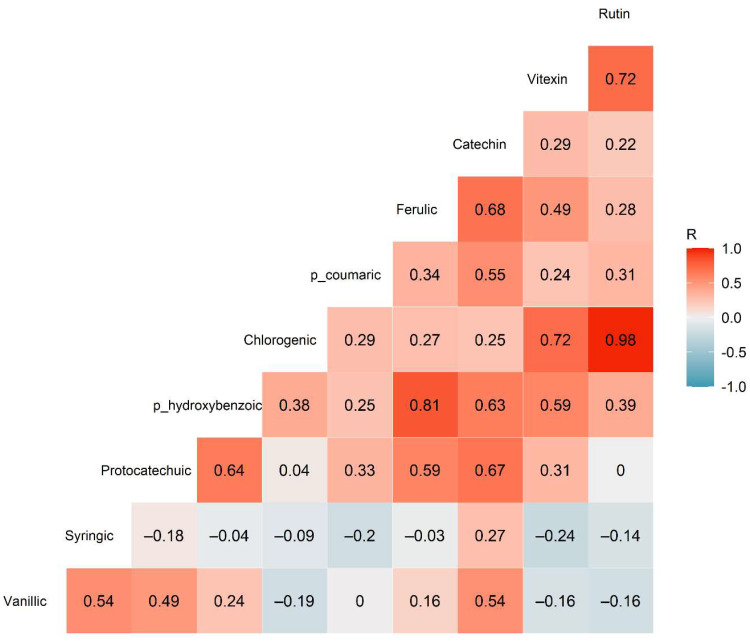
Correlogram showing the correlations between the various phenolic acids and flavonoids quantified in the faba bean samples (*n* = 60 replicates). The numbers inside each square show the Pearson R correlation values.

Finally, correlation analysis was performed between the 10 phenolic compounds to ascertain if the concentrations of any specific compounds were closely linked to the concentrations of another compound. This may occur due to similar synthesis pathways between the compounds [30] or result from regulatory genes controlling multiple synthesis pathways. The correlation results demonstrated moderate to strong correlations between several compounds, most notably between rutin and chlorogenic acid (r_60_ = 0.979, *p* < 0.001), and between ferulic and *p*-hydroxybenzoic acid (r_60_ = 0.812, *p* < 0.001) (Figure 4). Rutin is a quercetin glycoside, while chlorogenic acid is the ester of caffeic acid and quinic acid, hence these compounds are not closely structurally related. However, both can be synthesised through the phenylpropanoid pathway [31], suggesting that a regulatory gene may be responsible for the correlation between these compounds. Similarly, ferulic acid is a hydroxycinnamic acid, while *p*-hydroxybenzoic acid is a simple hydroxybenzoic acid; however, both can be produced through the shikimate biosynthesis pathway [32].

## 3. Materials and Methods

### 3.1. Seed Material

Faba bean seed material was sourced from growing field trials in South Australia, as previously described [33]. This comprised material from 10 varieties, each of which was grown at 2 different sites (Charlick and Freeling) during the 2017 season. Three within-field replicates (each comprising approximately 50 g) were subsampled from the mechanically harvested composite sample for each treatment [33], for a total of 60 samples. Whole seeds were coarsely ground (Van Gelder grinder with a 3 mm screen) before being finely ground to a fine flour (Falling Number grinder with 0.8 mm screen) [33].

### 3.2. Extraction of Phenolic Compounds

Polar phenolic compounds were extracted from the faba bean flour using 90% methanol, following previously reported methods [18]. The total phenolic content was determined using the Folin–Ciocalteu method, as previously described [18].

### 3.3. Phenolic Profiling by HPLC

Each methanol extract (20 mL) was concentrated using a rotary evaporator with the water bath temperature limited to 27 °C, before being reconstituted in 1 mL of methanol and syringe filtered (Livingstone 0.45 µm PTFE). The phenolics were separated on an Agilent 1100 HPLC system (Waldbronn, Germany) using previously described methods [27]. Briefly, a reversed-phase C_18_ column (Agilent Eclipse XDB-C18; 150 × 4.6 mm; 5 µm pore size) and guard cartridge (Gemini C_18_ 4 × 2 mm) were used, with an injection volume of 5 µL and column temperature of 27 ± 0.8 °C. The mobile phase comprised 0.01 M phosphoric acid (A) and methanol (B) at a flow rate of 1 mL/min, with the gradient beginning at 20% B and ramping linearly to reach 100% B at 20 min. The total run time was 25 min, with a post-run equilibration time of 7 min.

Compounds were identified based on a comparison of their retention time and UV spectra with authentic standards (Sigma Aldrich Australia). The purity of the peaks was confirmed by examination of the UV spectra at different points throughout the peak. Quality-of-analysis parameters are shown in Table 3.

### 3.4. Data Analysis

Statistical analysis was performed in R Studio running R 4.0.2 [34]. A two-way ANOVA was performed to assess the impact of variety and growing site on the content of various constituents. This was considered appropriate as the majority of data were approximately normally distributed and due to the large sample size (*n* = 60), the Central limit theorem could be applied to the dataset. Principal component analysis was performed in the Unscrambler X 10.5 software (Camo ASA, Oslo, Norway). Where applicable, results were presented as mean ± 1 standard deviation.

## 4. Conclusions

This study profiled the phenolic acid and flavonoid composition in 10 commercial varieties of Australian faba bean for the first time. The most abundant compounds were catechin and syringic acid, with rutin, vitexin, protocatechuic, vanillic, *p*-hydroxybenzoic, chlorogenic, *p*-coumaric, and *trans*-ferulic acid all found in low concentrations. The content of most individual phenolics varied significantly with the variety while growing location had a significant effect for around half of these compounds. Genotype × location interactions were only observed for vanillic, syringic, and *trans*-ferulic acids. Significant correlations were observed between a number of constituents, including between rutin and chlorogenic acid, and between ferulic and *p*-hydroxybenzoic acid. Notably, PBA Rana showed a distinct phenolic profile compared to the remaining nine varieties, supporting the findings of earlier research. In addition to providing baseline information on the typical phenolic contents of Australian-grown faba bean varieties, this study may inform plant breeders and growers in optimising the potential health benefits of the Australian faba bean crop.

## Figures and Tables

**Table 1:** Mean phenolic acid and flavonoid contents in the 10 faba bean varieties. Values given in µg/g (mean ± SD from 6 replicates, comprising within-field triplicates from two field locations). *p*-value indicates the significance between varieties, with results obtained from a two-way ANOVA between site × variety. Note that entries in the same row containing the same superscript letter (a–d) were not significantly different from one another at α = 0.05.

Compound	Doza	Farah	Fiesta VF	Fiord	Nura	PBA Nasma	PBA Rana	PBA Samira	PBA Warda	PBA Zahra	*p* Value
Protocatechuic acid	1.88 ± 0.83 ^b^	1.45 ± 0.61 ^b^	1.44 ± 0.50 ^b^	1.66 ± 0.77 ^b^	1.29 ± 0.22 ^b^	1.65 ± 0.60 ^b^	2.93 ± 1.07 ^a^	2.09 ± 0.77 ^ab^	1.83 ± 0.56 ^b^	1.81 ± 0.36 ^b^	***
*p*-hydroxybenzoic acid	0.57 ± 0.06 ^bcd^	0.52 ± 0.07 ^cd^	0.52 ± 0.11 ^cd^	0.64 ± 0.18 ^bcd^	0.44 ± 0.08 ^d^	0.62 ± 0.13 ^bcd^	1.11 ± 0.21 ^a^	0.73 ± 0.15 ^bc^	0.61 ± 0.10 ^bcd^	0.79 ± 0.11 ^b^	***
Vanillic acid	2.46 ± 0.59 ^ab^	1.87 ± 0.37 ^b^	1.96 ± 0.36 ^b^	1.88 ± 0.43 ^b^	2.40 ± 0.24 ^ab^	1.96 ± 0.29 ^b^	2.24 ± 0.68 ^ab^	2.81 ± 0.52 ^a^	2.71 ± 0.40 ^a^	2.76 ± 0.85 ^a^	***
Syringic acid	77.6 ± 11.2 ^c^	72.4 ± 7.9 ^c^	80.5 ± 13.1 ^c^	77.6 ± 11.8 ^c^	149.8 ± 14.8 ^a^	77.8 ± 9.5 ^c^	72.5 ± 7.3 ^c^	109.6 ± 21.6 ^b^	80.9 ± 14.1 ^c^	122.5 ± 14.4 ^b^	***
Sum of hydroxybenzoic acids	82.5 ± 12.6 ^c^	76.2 ± 8.5 ^c^	84.4 ± 13.7 ^c^	81.8 ± 12.7 ^c^	153.9 ± 14.9 ^a^	82.0 ± 10.3 ^c^	78.8 ± 8.8 ^c^	115.2 ± 22.8 ^b^	86.0 ± 14.8 ^c^	127.9 ± 15.4 ^b^	***
Chlorogenic acid	0.89 ± 0.96	0.85 ± 0.52	1.27 ± 1.30	2.88 ± 2.56	0.78 ± 0.41	0.89 ± 0.44	3.02 ± 3.31	1.14 ± 0.73	1.70 ± 2.48	1.98 ± 3.41	NS
*p*-coumaric acid	1.21 ± 0.21 ^bc^	1.64 ± 0.25 ^ab^	1.86 ± 0.40 ^a^	1.69 ± 0.28 ^ab^	1.26 ± 0.16 ^abc^	1.52 ± 0.37 ^abc^	1.70 ± 0.27 ^ab^	1.52 ± 0.38 ^abc^	1.62 ± 0.54 ^ab^	0.95 ± 0.17 ^c^	***
*trans*-ferulic acid	1.27 ± 0.22 ^b^	0.96 ± 0.21 ^b^	1.11 ± 0.27 ^b^	1.42 ± 0.42 ^b^	1.36 ± 0.32 ^b^	1.22 ± 0.35 ^b^	2.99 ± 0.65 ^a^	1.34 ± 0.25 ^b^	1.26 ± 0.30 ^b^	1.82 ± 0.18 ^b^	***
Sum of hydroxycinnamic acids	3.37 ± 1.09 ^b^	3.45 ± 0.76 ^b^	4.24 ± 1.81 ^ab^	5.99 ± 2.97 ^ab^	3.40 ± 0.68 ^b^	3.63 ± 0.85 ^ab^	7.71 ± 2.83 ^a^	4.00 ± 1.17 ^ab^	4.58 ± 3.22 ^ab^	4.11 ± 3.60 ^ab^	*
Catechin	216 ± 64 ^ab^	191 ± 37 ^b^	215 ± 52 ^ab^	245 ± 52 ^ab^	232 ± 27 ^ab^	207 ± 37 ^b^	297 ± 53 ^a^	240 ± 55 ^ab^	258 ± 63 ^ab^	220 ± 33 ^ab^	*
Vitexin	0.88 ± 0.24 ^b^	1.70 ± 1.82 ^ab^	0.97 ± 0.39 ^b^	1.52 ± 1.82 ^ab^	0.58 ± 0.41 ^b^	0.80 ± 0.28 ^b^	3.50 ± 1.43 ^a^	0.75 ± 0.43 ^b^	1.21 ± 0.82 ^b^	1.43 ± 1.52 ^ab^	**
Rutin	5.55 ± 5.02	7.34 ± 5.11	7.66 ± 6.36	13.91 ± 11.81	4.04 ± 3.00	4.50 ± 2.30	15.87 ± 14.22	7.67 ± 4.09	10.48 ± 10.60	9.43 ± 16.29	NS
Sum of flavonoids	223 ± 61 ^ab^	200 ± 37 ^b^	223 ± 57 ^ab^	261 ± 61 ^ab^	237 ± 28 ^ab^	212 ± 37 ^b^	316 ± 45 ^a^	248 ± 56 ^ab^	269 ± 71 ^ab^	231 ± 35 ^ab^	*

NS = not significant (*p* > 0.05), * *p* < 0.05, ** *p* < 0.01, *** *p* < 0.001.

**Table 2:** Impact of the growing site on phenolic acid and flavonoid contents. Values given in µg/g (mean ± SD from 3 replicates for each location). The *p*-value column indicates the significance between sites, with results obtained from a two-way ANOVA between site × variety.

Compound	Charlick (*n* = 30)	Freeling (*n* = 30)	Site *p* Value	Variety × Site Interaction
Protocatechuic acid	1.43 ± 0.36	2.17 ± 0.87	***	NS
*p*-hydroxybenzoic acid	0.67 ± 0.19	0.64 ± 0.25	NS	NS
Vanillic acid	2.11 ± 0.41	2.50 ± 0.67	***	**
Syringic acid	89.3 ± 28.2	94.9 ± 27.9	NS	*
Sum of hydroxybenzoic acids	93.5 ± 28.3	100.2 ± 28.4	*	*
Chlorogenic acid	2.22 ± 2.57	0.86 ± 0.68	**	NS
*p*-coumaric acid	1.45 ± 0.42	1.55 ± 0.38	NS	NS
*trans*-ferulic acid	1.45 ± 0.47	1.38 ± 0.77	NS	**
Sum of hydroxycinnamic acids	5.11 ± 3.00	3.78 ± 1.39	*	NS
Catechin	224 ± 45	240 ± 60	NS	NS
Vitexin	1.67 ± 1.56	0.98 ± 0.86	*	NS
Rutin	12.21 ± 11.59	5.07 ± 3.54	**	NS
Sum of flavonoids	238 ± 52	246 ± 62	NS	NS

* *p* < 0.05, ** *p* < 0.01, *** *p* < 0.001

**Table 3:** Quality-of-analysis parameters for the phenolic standards. All calibrations were performed at concentrations between 1–100 mg L^−1^.

No.	Compound	Retention Time (min)	Wavelength (nm)	Slope	LOD (mg L^−1^)	LOQ (mg L^−1^)	Calibration R^2^
	Hydroxybenzoic acids						
1	Protocatechuic acid	3.94	250 nm	13.5	0.1	0.4	1
4	*p*-hydroxybenzoic acid	5.78	250 nm	25.9	0.1	0.2	1
5	Vanillic acid	6.26	250 nm	12.3	0.1	0.4	1
6	Syringic acid	6.59	280 nm	15.1	0.1	0.3	1
	Hydroxycinnamic acids						
3	Chlorogenic acid	5.26	320 nm	14.1	0.1	0.4	1
7	*p*-coumaric acid	8.12	320 nm	32.3	0.05	0.2	1
9	*trans*-ferulic acid	8.44	320 nm	26.9	0.1	0.2	1
	Flavonoids						
2	Catechin	4.55	280 nm	4.0	0.4	1.3	1
8	Vitexin	8.17	320 nm	7.3	0.2	0.7	0.9999
10	Rutin	9.82	250 nm	6.2	0.2	0.8	1

## Data Availability

The data presented in this study are available on request from the corresponding author.
